# Analyzing bifurcation, stability, and wave solutions in nonlinear telecommunications models using transmission lines, Hamiltonian and Jacobian techniques

**DOI:** 10.1038/s41598-024-64788-w

**Published:** 2024-07-03

**Authors:** Ahmed Refaie Ali, Harun Or Roshid, Shariful Islam, Asma Khatun

**Affiliations:** 1 Department of Mathematics and Computer Science, Faculty of Science, Menoufia University, Menofia Governorate, Shibin El-Kom, 32511 Egypt; 2 Department of Mathematics, Pabna University of Science and Technology, Pabna, 6600 Bangladesh; 3 Department of Electrical and Electronic Engineering, Pundra University of Science and Technology, Bogura, 5800 Bangladesh; 4 Department of Mathematics, Sunamgonj Science and Technology University, Sunamganj, Sunamganj, Sylhet 3000 Bangladesh

**Keywords:** Wave propagation, Electrical transmission lines, NPDEs, Travelling wave, Modified expansion, Jacobi elliptic, Computational science, Applied mathematics, Energy grids and networks, Power distribution

## Abstract

This study presents a comprehensive analysis of a nonlinear telecommunications model, exploring bifurcation, stability, and wave solutions using Hamiltonian and Jacobian techniques. The investigation begins with a thorough examination of bifurcation behavior, identifying critical points and their stability characteristics, leading to the discovery of diverse bifurcation scenarios. The stability of critical points is further assessed through graphical and numerical methods, highlighting the sensitivity to parameter variations. The study delves into the derivation of both numerical and analytical wave solutions, aligning them with energy orbits depicted in phase portraits, revealing a spectrum of wave behaviors. Additionally, the analysis extends to traveling wave solutions, providing insights into wave propagation dynamics. Notably, the study underscores the efficacy of the planar dynamical approach in capturing system behavior in harmony with phase portrait orbits. The findings have significant implications for telecommunications engineers and researchers, offering insights into system behavior, stability, and signal propagation, ultimately advancing our understanding of complex nonlinear dynamics in telecommunications networks.

## Introduction

### Related work

Modern research into the causes of physical issues, spanning fields such as communication systems, fluid dynamics, mathematics, physics, quantum field theory, and nonlinear fiber optics, heavily relies on nonlinear partial differential equations (NPDEs). Additionally, NPDE systems are increasingly regarded as vital tools for research in both chemical, telecommunication technology and biological studies. In the literature exploring the evolution of communication methods, several key works stand out. Brockedone’s^1^ detailed study on the development of the electric telegraph marks a significant technological leap in human connectivity. Levi’s^2^ exploration into the historical role of pigeons in communication provides a unique perspective on avian contributions to this field. Meanwhile, Blechman^3^ delves into the complex and multifaceted history of pigeons, illustrating their varied roles in human society. These works^1–3^ collectively offer a diverse and in-depth look at the progression of communication techniques, from groundbreaking inventions to the natural world's contributions. Various methods have been presented in the literature to address these NPDEs, including the extended tanh method, the sin-Gordan expansion method, the inverse scattering transform method, the extended mean value theorem, the tanh and extended tanh methods, the Jacobi elliptic function expansion method^8^, the modified expansion method, the generalized tanh methods, the finite difference method, and the interval-valued fuzzy topics method^8,9,20–22^. Cordero et al. have examined the stability of the fourth-order iterative method for the (2 + 1)-dimensional generalized modified dispersive water wave equation^4^, while extensive research has been conducted on bifurcation analysis and precise traveling wave solutions^4^. Travelling wave solutions to the nonlinear Klein Gordon equation have also been noted^10–19^ in various sources^20–22^ and elsewhere^5–7^.

Further investigations have explored electromagnetic wave phenomena in various mediums^18,20–34^. For instance, Abo-Seida et al. studied the influence of diamond and silver as cavity resonator wall materials on resonant frequency^25^. Abo-Seida et al.^27^ delve into Cherenkov Free-Electron Laser reactions in plasma-filled cylindrical waveguides within fractional D-dimensional space. El-Dabe and colleagues^28^ investigate the effects of thermophoresis on unsteady magnetohydrodynamic (MHD) flow of a radiation-absorbing fluid. On the other hand, Islam, Halder, and Refaie Ali^29^ provide insights into optical and rogue soliton solutions of the (2 + 1)-dimensional nonlinear Heisenberg ferromagnetic spin chains equation. Refaie Ali and co-authors^30^ explore electromagnetic wave propagation in plasma-filled rectangular waveguides using fractional space and LFD. Yang et al.^31^ introduce an even entire function as a special solution for a classical wave equation. Smolyaninov and Kozyrev^32^ contribute to the field of electromagnetic wave propagation through stratified lossy conductive media. Mahmuda Maya et al.^33^ investigate the influence of a magnetic field on MHD mixed convection in a lid-driven cavity with a wavy bottom surface. Bhrawy et al.^34^ delve into soliton and wave solutions of the Whitham–Broer–Kaup system, while Gatea et al.^35^ investigate the optical properties of ferroelectric thin films. Gheisari and Ong^36^ study the magnetization behavior of nanocrystalline Permalloy thin films, and Jayamurugan et al.^37^ investigate the optical, morphological, and thermal properties of spray-coated polypyrrole films. Mohamed and Hadia^38^ explore the influence of post-thermal annealing on the optical properties of SnO2 films prepared by electron beam evaporation. The referenced literature covers various aspects of thin films and wave systems. Bhrawy et al.^34^ investigated solitons, cnoidal waves, snoidal waves, and other solutions to the Whitham–Broer–Kaup system. Gatea et al.^35^ explored the optical properties of ferroelectric thin films prepared through the PLD technique. Gheisari and Ong^36^ focused on the magnetization behavior of nanocrystalline Permalloy thin films using oblique-angle magnetron sputtering. Jayamurugan et al.^37^ conducted an investigation into the optical, morphological, and thermal properties of spray-coated polypyrrole films. Lastly, Mohamed and Hadia^38^ examined the influence of post-thermal annealing on the optical properties of SnO2 films prepared via electron beam evaporation. These studies collectively contribute valuable insights into the properties and applications of thin films and wave systems across different research domains. Helal et al.^39^ examine wave propagation over a beach within a nonlinear theory, and Abdel-Gawad and Tantawy^40^ provide exact solutions of the Shamel-Korteweg-de Vries equation with time-dependent coefficients. These papers^27–42^ collectively contribute to our understanding of various physical and mathematical phenomena across different disciplines, enhancing our knowledge of these complex phenomena. El-Dabe et al.^41^ conducted research on hall currents and thermophoresis effects^41^, and they also explored the influence of thermophoresis on unsteady MHD Kuvshinski model^42^. Zendehnam et al.^43^ investigated the statistical surface morphology and optical properties of thin double-layers made of Ag/Al and Ag/Cu. Iqbal et al.^44^ explored bifurcation features, chaos, and coherent structures in one-dimensional nonlinear electrical transmission lines. Additionally, Islam, Khan, and Akbar^45^ examined optical soliton solutions, bifurcation, and stability analysis in the Chen-Lee-Liu model.

The recent advancements in the field of optical soliton solutions for various nonlinear equations have been significantly contributed to by the works of Ghanbari and collaborators^46–54^. Ghanbari and Gómez-Aguilar^46^ made notable strides in this area by exploring optical soliton solutions for the nonlinear Radhakrishnan-Kundu-Lakshmanan and Schrödinger equations with second-order spatio-temporal dispersion, highlighting innovative approaches to understanding these complex phenomena (Ghanbari & Gómez‐Aguilar^46,47^). Ghanbari's collaboration with Băleanu^48^ introduced new optical solutions for the fractional Gerdjikov-Ivanov equation, employing conformable derivatives. Additionally, Khater and Ghanbari's research^49^ provided insight into the solitary wave solutions and physical characterization of gas diffusion, furthering the understanding of these dynamics in homogeneous mediums. Ghanbari et. al^50–54^ have significantly contributed to the field, offering a plethora of soliton solutions for various equations like the Hirota-Maccari, Benjamin-Bona-Mahony, and Schamel equations, employing methods like the generalized exponential rational function method. This body of work not only enhances our understanding of these complex equations but also paves the way for further research in the field.

In recent scholarly contributions to the field of optical and quantum electronics, significant advancements have been made. Ullah, Roshid, and Ali (2024) explored new wave behaviors and conducted a comprehensive stability analysis of the (2 + 1)-dimensional Zoomeron model^23^. Roshid, et al. (2024), have delved into the stability and spin solitonic dynamics of the HFSC model, focusing on the effects of neighboring interactions and crystal field anisotropy parameters^24^. These studies^23,24^ collectively enhance our understanding of nonlinear dynamics and their applications in telecommunications.

### Motivations and justification

We understand the importance of providing a clear rationale for the research and its significance. Allow us to provide the motivations and justification for considering the problem in our study:

1. Relevance to Telecommunications Industry: The telecommunications industry plays a pivotal role in our modern interconnected world. As such, it faces various challenges in ensuring the efficient and reliable transmission of signals over electrical transmission lines. Nonlinear effects within these lines can lead to signal distortion and instability, making it crucial to understand and mitigate such issues. Our study addresses a nonlinear telecommunications model to offer insights that are directly relevant to this industry.
2. Complexity of Nonlinear Dynamics: Nonlinear dynamics are inherently complex and can lead to a wide range of behaviors, including bifurcations, chaotic dynamics, and wave propagation phenomena. The choice of this problem allows us to explore the intricate behavior of nonlinear systems, contributing to the understanding of complex phenomena that may arise in real-world telecommunications networks.
3. Scientific and Engineering Significance: Analyzing the bifurcation, stability, and wave solutions in this nonlinear model provides valuable scientific and engineering insights. It offers the opportunity to predict and manage system transitions and instabilities, optimize network stability, and design communication systems capable of accommodating a variety of signal types and characteristics.
4. Novelty in Methodology: Our study introduces a novel approach by combining Hamiltonian and Jacobian techniques within a planar dynamical framework. This integration offers a fresh perspective on the analysis of nonlinear telecommunications models and has the potential to advance the methodology used in similar studies.
5. Contribution to Knowledge: By delving into this problem, we aim to contribute to the body of knowledge in both nonlinear dynamics and telecommunications. We believe that our comprehensive analysis and insights have the potential to enhance the understanding of complex systems in this field.

### Novelty and contributions of the paper

1. *Comprehensive analysis:* Our paper presents a comprehensive analysis of a nonlinear telecommunications model, which encompasses bifurcation, stability, and wave solutions. The novelty lies in the integration of these diverse aspects into a unified framework, offering a holistic understanding of the system’s behavior.
2. *Integration of Hamiltonian and Jacobian techniques:* We employ a combination of Hamiltonian and Jacobian techniques, which is a novel approach in the context of nonlinear telecommunications models. This integration allows us to explore the system’s dynamics in depth, providing unique insights into its behavior. See (Fig. 4.2, 4.3, 4.4)
3. *Energy orbits in phase portraits:* Our research introduces the concept of energy orbits depicted in phase portraits, which is a novel visualization tool. This novel approach helps readers interpret the energy-related aspects of the system's behavior, offering a fresh perspective on nonlinear dynamics.
4. *Diverse wave solutions:* We derive a wide range of wave solutions, including solitons, periodic waves, and nonlinear waveforms. This diversity in wave solutions is a significant contribution, as it demonstrates the model's adaptability in representing different signal propagation scenarios.
5. *Traveling wave insights:* Our analysis extends to traveling wave solutions within the model, providing valuable insights into wave propagation dynamics. This contribution enhances our understanding of signal transmission over long distances and through complex networks.
6. *Planar dynamical approach:* We highlight the efficacy of the planar dynamical approach, which offers a unique and effective way to capture system behavior in alignment with phase portrait orbits. This approach contributes to a deeper understanding of the model’s dynamics.

Our paper’s novelty and contributions lie in the comprehensive analysis of a nonlinear telecommunications model, the integration of Hamiltonian and Jacobian techniques, the introduction of energy orbits in phase portraits, the derivation of diverse wave solutions, insights into traveling wave behavior, and the effectiveness of the planar dynamical approach. These elements collectively advance our understanding of complex systems in telecommunications and nonlinear dynamics, making our research a valuable addition to the field.

The nonlinear electrical transmission line has several novel complicated dark, trigonometric, and hyperbolic solitons introduced through mathematical analysis.

The following equation, as defined in^9^, has been proposed to represent physical features in terms of mathematical dynamics, such as the wave propagation of electrical transmission lines:

1 $$u_{tt} - \mu (u^{2} )_{tt} + \rho (u^{3} )_{tt} - \varepsilon_{0}^{2} \lambda_{1}^{2} u_{xx} - \varepsilon_{0}^{2} \frac{{\lambda_{1}^{4} }}{12}u_{xxxx} - \theta_{0}^{2} \lambda_{2}^{2} u_{yy} - \theta_{0}^{2} \frac{{\lambda_{2}^{2} }}{12}u_{yyyy} = 0,$$

where $u = u(x,y,t)$ is used to explain the tightness throughout the electrical line and $x$ and $y$ are interpreted like the promulgation distance. $t$ is the period, and $\mu$ and $\rho$ are constants with nonzero. $\lambda_{1}$ is the space between two proximate sections in during longitudinally flank, while $\lambda_{2}$ is the space between two proximate sections in the transversal flank^9^. With the assistance of computational programs, we can generate theoretical results and represent them through 2D, 3D, and contour surface plots.

## Problem formulation

### Mathematical analysis

We define the traveling wave transformation as follows:

2 $$u = \nu (\eta ),\;\eta = p(x + y - kt),$$

where $p,k$ are nonzero constants or complex-valued parameters. When we incorporate Eq. (2) into Eq. (1), we obtain:

3 $$k^{2} \nu_{\eta \eta } - \mu k^{2} (\nu^{2} )_{\eta \eta } + \rho k^{2} (\nu^{3} )_{\eta \eta } - \varepsilon_{0}^{2} \lambda_{1}^{2} \nu_{\eta \eta } - p^{2} \varepsilon_{0}^{2} \frac{{\lambda_{1}^{4} }}{12}\nu_{\eta \eta \eta \eta } - \theta_{0}^{2} \lambda_{2}^{2} \nu_{\eta \eta } - p^{2} \theta_{0}^{2} \frac{{\lambda_{2}^{2} }}{12}\nu_{\eta \eta \eta \eta } = 0.$$

Integrating Eq. (3) twice with respect to $\eta$, setting the constants of integrations to zero yields

4 $$12\left[ {k^{2} - \varepsilon_{0}^{2} \lambda_{1}^{2} - \theta_{0}^{2} \lambda_{2}^{2} } \right]\nu - 12\mu k^{2} \nu^{2} + 12\rho k^{2} \nu^{3} - p^{2} \left[ {\varepsilon_{0}^{2} \lambda_{1}^{4} + \theta_{0}^{2} \lambda_{2}^{4} } \right]\nu^{\prime\prime} = 0$$

5 $$\Rightarrow P\nu^{\prime\prime} = Q\nu - R\nu^{2} + S\nu^{3}$$

where,

$$P = p^{2} (\varepsilon_{0}^{2} \lambda_{1}^{4} + \theta_{0}^{2} \lambda_{2}^{4} )$$

$$Q = 12(k^{2} - \varepsilon_{0}^{2} \lambda_{1}^{2} - \theta_{0}^{2} \lambda_{2}^{2} )$$

$$R = 12\mu k^{2}$$

$$S = 12\rho k^{2}$$

### Critical points

Let, $$\nu^{\prime} = U$$

$$\Rightarrow U^{\prime} = \frac{Q}{P}\nu - \frac{R}{P}\nu^{2} + \frac{S}{P}\nu^{3}$$

Critical point $$U = 0$$

$$\frac{Q}{p}\nu - \frac{R}{p}\nu^{2} + \frac{S}{P}\nu^{3} = 0$$

$$\Rightarrow \nu (Q - R\nu + S\nu^{2} ) = 0$$

$$\therefore \nu = 0,\;\;\;\;\nu = \frac{R}{2S} \pm \frac{{\sqrt {R^{2} - 4QS} }}{2S}$$

Hence we get three critical points,

$$(\nu ,U) = (0,0),\left( {\frac{R}{2S} + \frac{{\sqrt {R^{2} - 4QS} }}{2S},0} \right),\left( {\frac{R}{2S} - \frac{{\sqrt {R^{2} - 4QS} }}{2S},0} \right)$$

## Hamiltonian and Jacobian of the system

Hamiltonian

$$H = \frac{{U^{2} }}{2} - \frac{Q}{2P}\nu^{2} + \frac{R}{3P}\nu^{3} - \frac{S}{4P}\nu^{4}$$

Let, $$f\left( {\nu ,U} \right) = U$$

$$g\left( {\nu ,U} \right) = \frac{Q}{P}\nu - \frac{R}{P}\nu^{2} + \frac{S}{P}\nu^{3}$$

Jacobian

$$J = \left| {\begin{array}{*{20}c} {f_{\nu } } & {f_{U} } \\ {g_{\nu } } & {g_{U} } \\ \end{array} } \right|$$

$$= \left| {\begin{array}{*{20}c} 0 & 1 \\ {\frac{Q}{P} - \frac{2R\nu }{P} + \frac{{3S\nu^{2} }}{P}} & 0 \\ \end{array} } \right|$$

Now,

$$J_{{\left( {0,0)} \right)}} = \left| {\begin{array}{*{20}c} 0 & 1 \\ \frac{Q}{P} & 0 \\ \end{array} } \right|$$

$$J_{{\left( {\frac{R}{2S} + \frac{{\sqrt {R^{2} - 4QS} }}{2S}.0} \right)}} = \left| {\begin{array}{*{20}c} 0 & 1 \\ {\frac{{R^{2} + R\sqrt {R^{2} - 4QS} - 4QS}}{2PS}} & 0 \\ \end{array} } \right|$$

and

$$J_{{\left( {\frac{R}{2S} - \frac{{\sqrt {R^{2} - 4QS} }}{2S}.0} \right)}} = \left| {\begin{array}{*{20}c} 0 & 1 \\ {\frac{{R^{2} - R\sqrt {R^{2} - 4QS} - 4QS}}{2PS}} & 0 \\ \end{array} } \right|$$

## Stability of the critical points with graphs and numerical solutions

**Case 1:** Specific choices for $\varepsilon_{0} = \theta_{0} = \lambda_{1} = \lambda_{2} = p = 1,\,k = 1,\mu = 1/12,\rho = 1/4$ implies $P = 2,Q = - 12,R = 1,S = 3$.

Then,

$$J_{{\left( {0,0)} \right)}} = \left| {\begin{array}{*{20}c} 0 & 1 \\ { - 6} & 0 \\ \end{array} } \right|$$

Here $\lambda = \pm \sqrt 6 i$. So the point is stable.

$$J_{{\left( {2.1736,0)} \right)}} = \left| {\begin{array}{*{20}c} 0 & 1 \\ {13.086} & 0 \\ \end{array} } \right|$$

Here $\lambda = \pm \sqrt {13.086}$. So the point is unstable.

$$J_{{\left( { - 1.8402,0)} \right)}} = \left| {\begin{array}{*{20}c} 0 & 1 \\ {11.0798} & 0 \\ \end{array} } \right|$$

Here $\lambda = \pm \sqrt {11.0798}$. So the point is unstable.
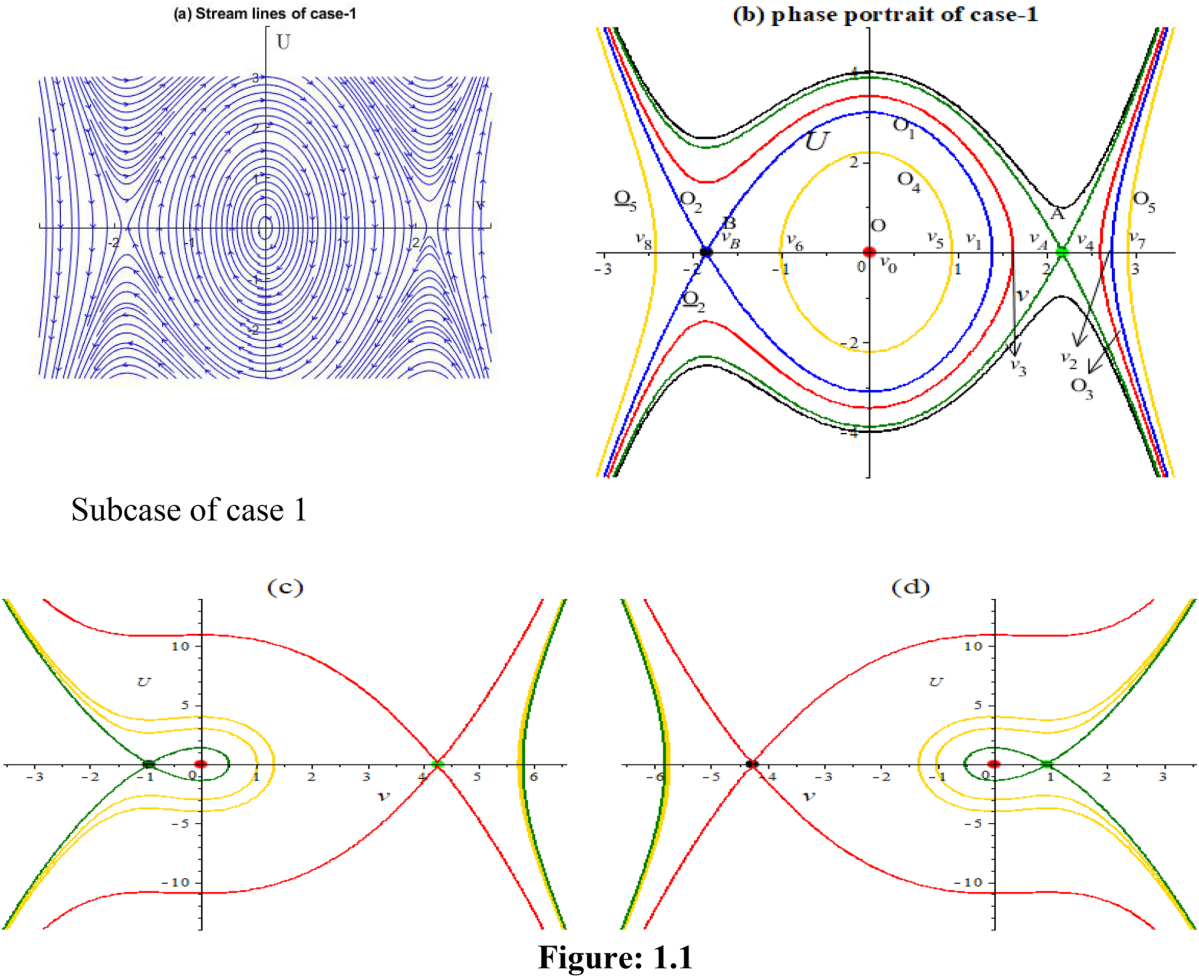


We obtain four numerical solutions for this case, each corresponding to a different set of initial points. The initial points we use are: (2.1736, 0), (-1.84, 0), (2.174, -1), and (1, 0). To realize the accuracy of solutions corresponding phase orbits are plotted also.
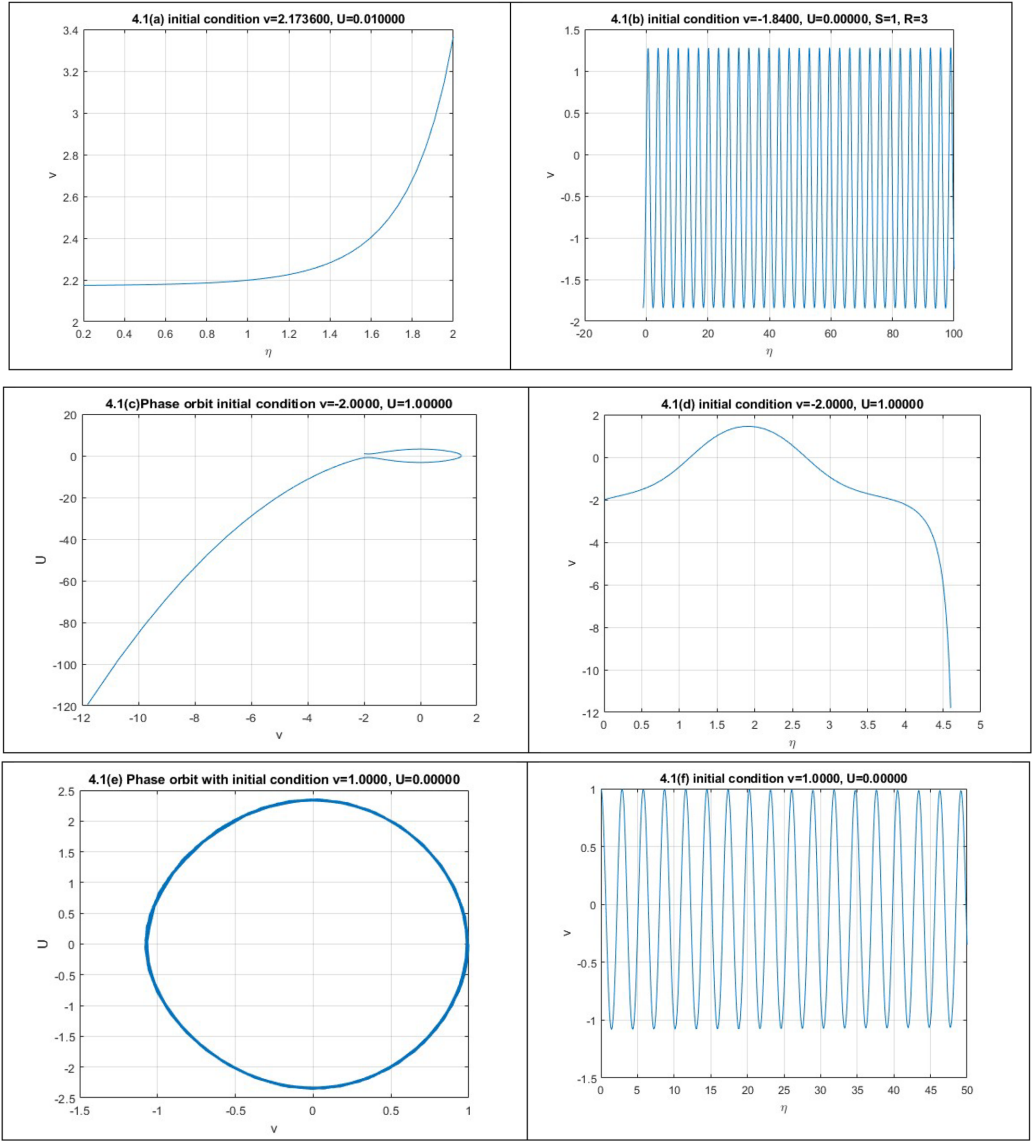


**Case-2:** Let $\varepsilon_{0} = \theta_{0} = \lambda_{1} = \lambda_{2} = p = 1,\,k = 5/\sqrt {12} ,\mu = 2/25,\rho = 1/25$ implies $P = 2,Q = 1,R = 2,S = 1$. Then,

$$J_{{\left( {0,0)} \right)}} = \left| {\begin{array}{*{20}c} 0 & 1 \\ {0.5} & 0 \\ \end{array} } \right|$$

Here $\lambda = \pm \sqrt {0.5}$. So the point is unstable.

$$J_{{\left( {1,0)} \right)}} = \left| {\begin{array}{*{20}c} 0 & 1 \\ 0 & 0 \\ \end{array} } \right|$$

Here $\lambda = 0,0$. So the point is unstable.

$$J_{{\left( {1,0)} \right)}} = \left| {\begin{array}{*{20}c} 0 & 1 \\ 0 & 0 \\ \end{array} } \right|$$

Here $\lambda = 0,0$. So the point is unstable.
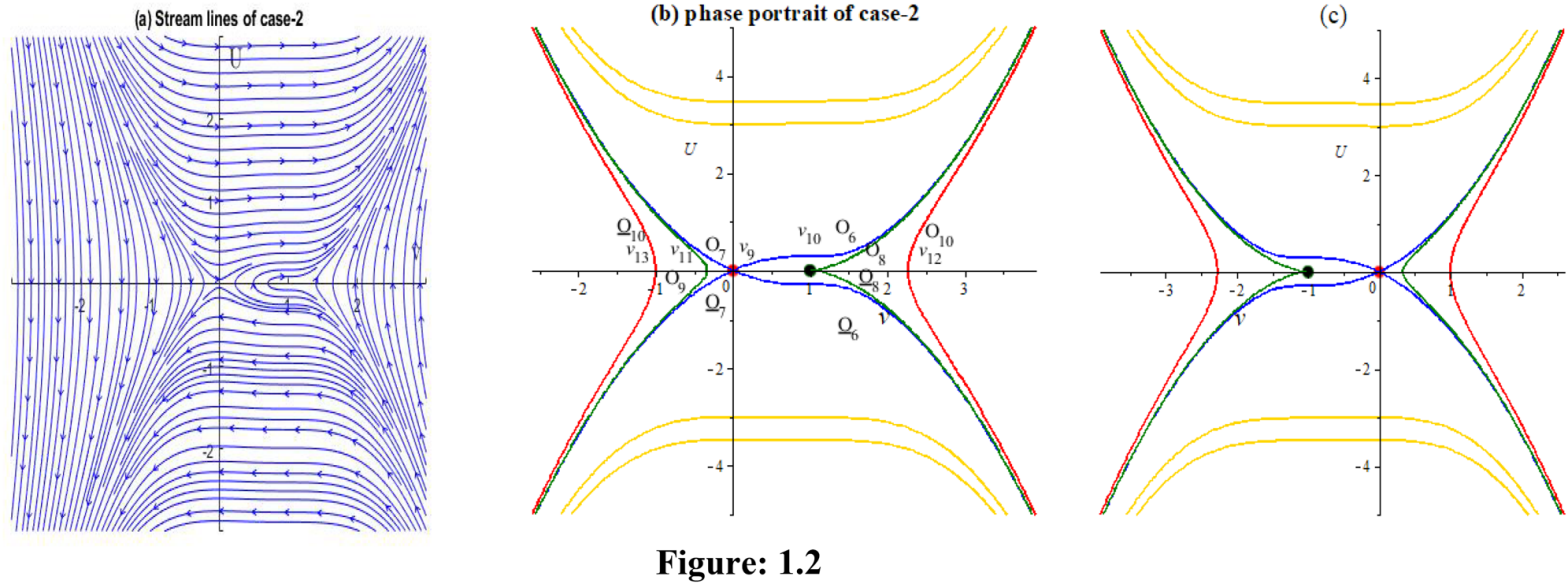


In this case, we obtain two numerical solutions by setting two distinct initial points: (0, 0.0001) and (-0.5, 0). Each initial point leads to a different type of solution.
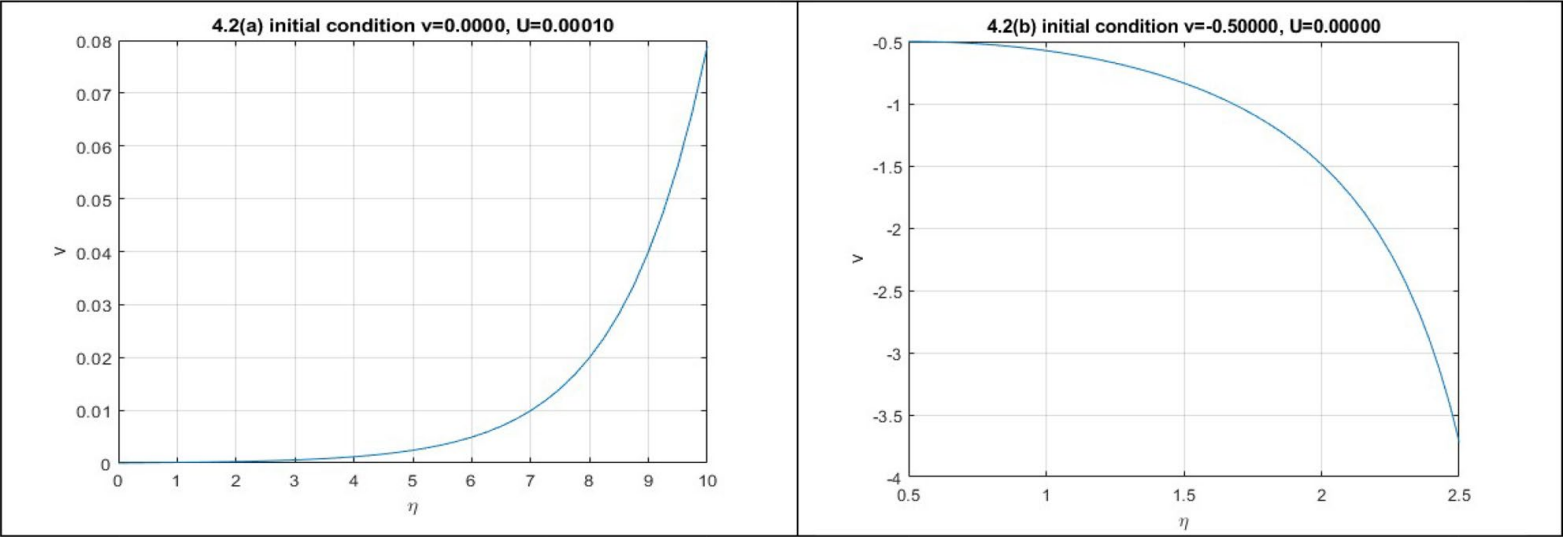


**Case 3:** In Fig. 1.3, let, $\varepsilon_{0} = \theta_{0} = \lambda_{1} = \lambda_{2} = 1,p = 2,\,k = 3,\mu = - 1/12,\rho = - 1/4$ implies $P = 8,Q = 84,R = - 9,S = - 27$. Then,

$$J_{{\left( {0,0)} \right)}} = \left| {\begin{array}{*{20}c} 0 & 1 \\ {10.5} & 0 \\ \end{array} } \right|$$

Here $\lambda = \pm \sqrt {10.5}$. So the point is unstable.

$$J_{{\left( { - 1.6050,0)} \right)}} = \left| {\begin{array}{*{20}c} 0 & 1 \\ { - 19.194} & 0 \\ \end{array} } \right|$$

Here $\lambda = \pm \sqrt {19.194} i$. So the point is stable.

$$J_{{\left( {1.9384,0)} \right)}} = \left| {\begin{array}{*{20}c} 0 & 1 \\ { - 23.180} & 0 \\ \end{array} } \right|$$

Here $\lambda = \pm \sqrt {23.180} i$. So the point is stable.
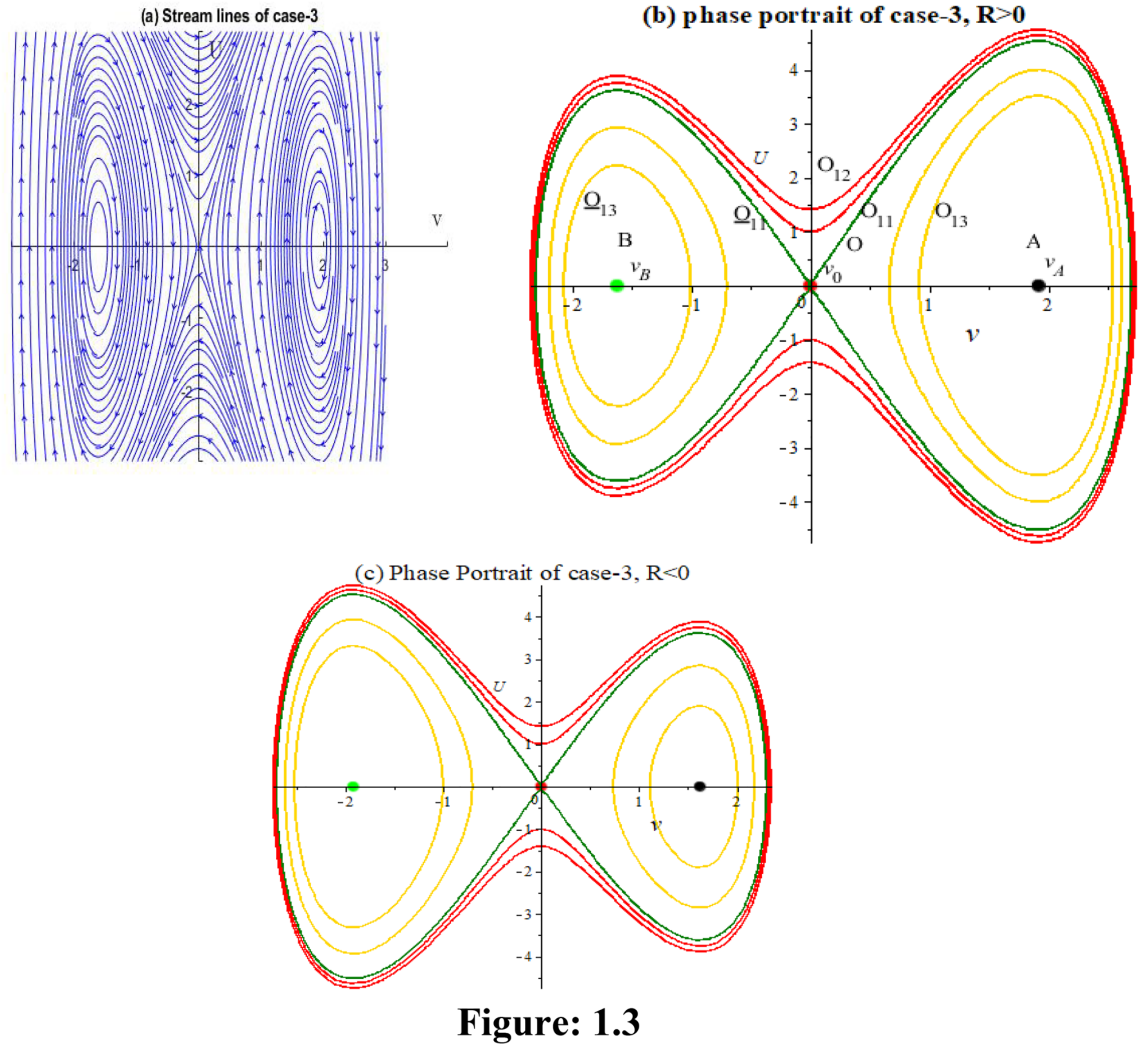


We obtain five numerical solutions for this case, as shown in Fig. 1.3, using five different initial points: (1.938, 0), (0, 0.2), (0.2, 0), (−0.2, 0), and (−0.3543, 0.07955) to get five types of solutions. To realize the accuracy of solutions corresponding phase orbits are plotted also.
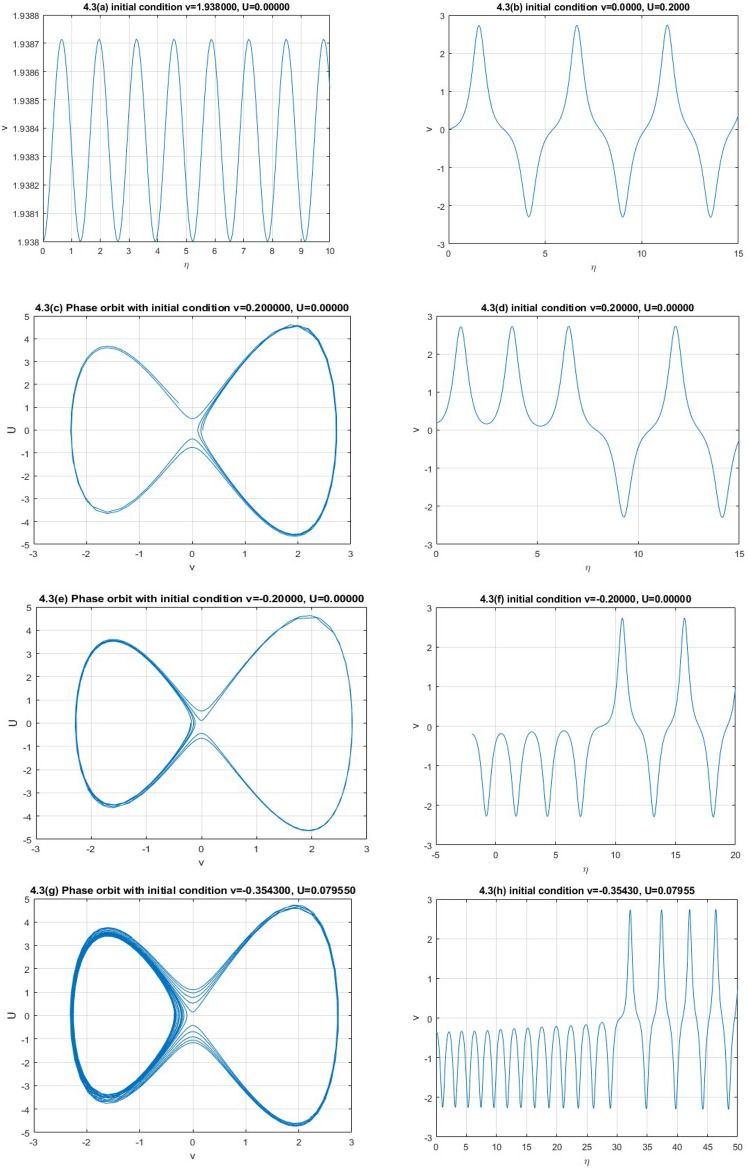


**Case 4:** In Fig. 1.4, let, $\varepsilon_{0} = \theta_{0} = \lambda_{1} = \lambda_{2} = 1,p = 2,\,k = 3,\mu = - 1/12,\rho = 1/4.$ implies $P = 8,Q = 84,R = - 9,S = 27$. Then,

$$J_{{\left( {0,0)} \right)}} = \left| {\begin{array}{*{20}c} 0 & 1 \\ {10.5} & 0 \\ \end{array} } \right|$$

Here $\lambda = \pm \sqrt {10.5}$. So, the point is unstable.

$$J_{{\left( { - 0.16666 + 1.7559i,0)} \right)}} = \left| {\begin{array}{*{20}c} 0 & 1 \\ { - 20.8125 - 1.9754i} & 0 \\ \end{array} } \right|$$

Here $\lambda = \pm i\sqrt {20.8125 + 1.9754i}$. So, the point is stable.

$$J_{{\left( { - 0.16666 - 1.7559i,0)} \right)}} = \left| {\begin{array}{*{20}c} 0 & 1 \\ { - 20.8125 + 1.9754i} & 0 \\ \end{array} } \right|$$

Here $\lambda = \pm i\sqrt {20.8125 - 1.9754i}$. So, the point is stable.
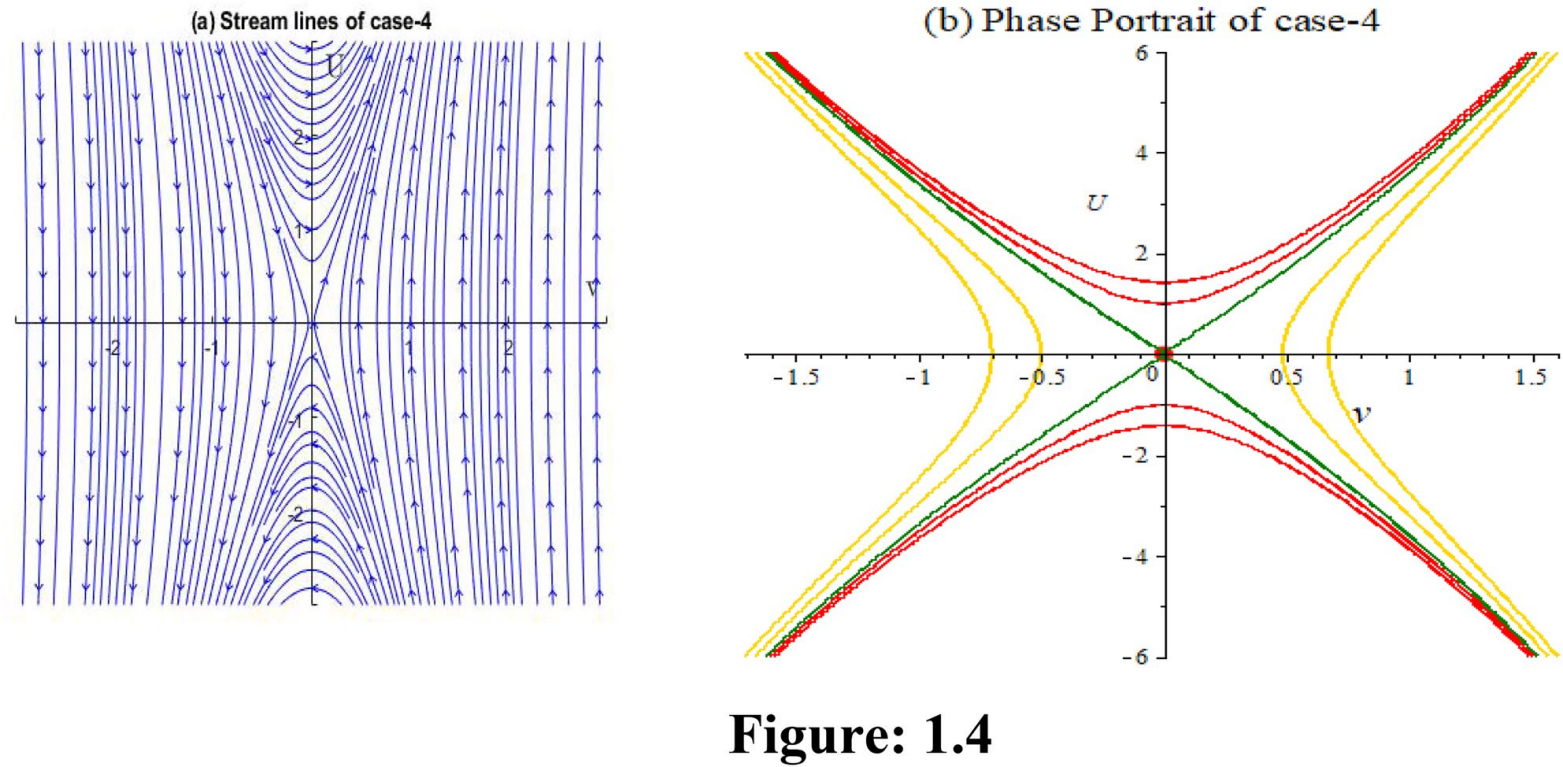


In Fig. 1.4, we obtain two numerical solutions for this case by using two different initial points, resulting in two distinct types of solutions. The initial points we employ are (-0.1667, 0) and (0.52371, -0.0806).
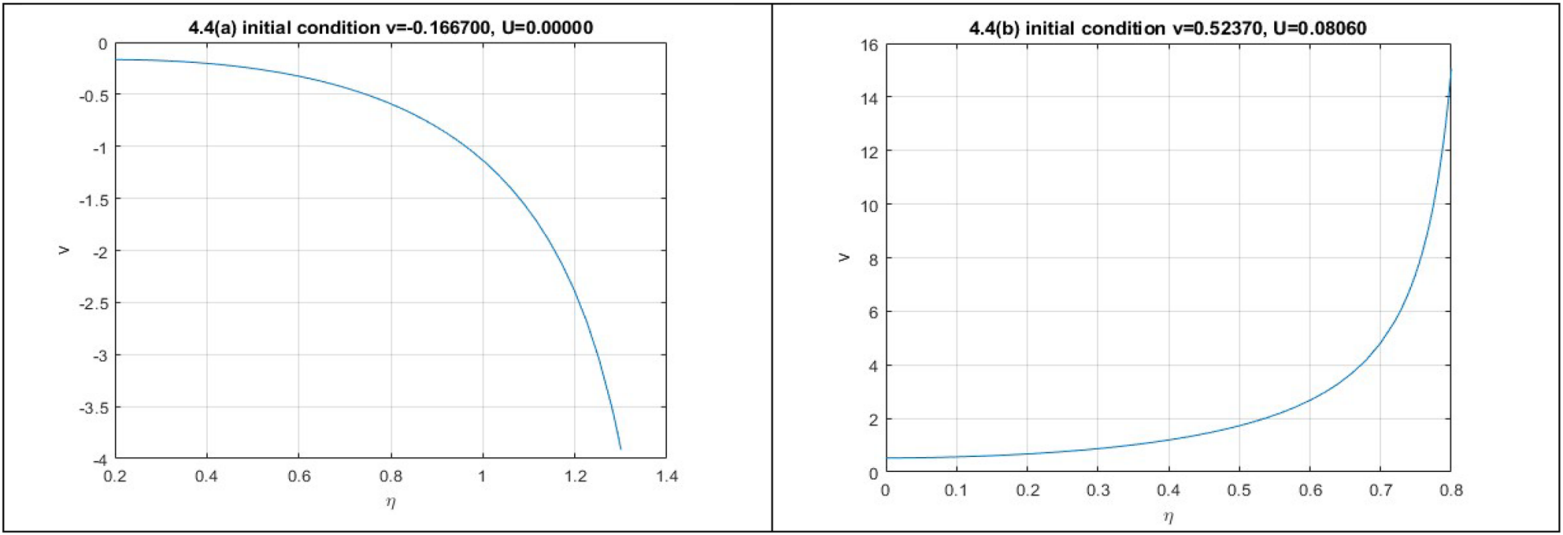


**Case 5:** In Fig. 1.5, let, $\varepsilon_{0} = \theta_{0} = \lambda_{1} = \lambda_{2} = 1,p = 2,\,k = 1,\mu = - 1/12,\rho = - 1/4$ implies $P = 8,Q = - 12,R = - 1,S = - 3$. Then,

$$J_{{\left( {0,0)} \right)}} = \left| {\begin{array}{*{20}c} 0 & 1 \\ { - 1.5} & 0 \\ \end{array} } \right|$$

Here $\lambda = \pm \sqrt {1.5} i$. So, the point is stable.

$$J_{{\left( {0.16666 - 1.9930i,0)} \right)}} = \left| {\begin{array}{*{20}c} 0 & 1 \\ {2.9791 + 0.2491i} & 0 \\ \end{array} } \right|$$

Here $\lambda = \pm \sqrt {2.9791 + 0.2491i}$. So, the point is unstable.

$$J_{{\left( {0.16666 + 1.9930i,0)} \right)}} = \left| {\begin{array}{*{20}c} 0 & 1 \\ {2.9791 - 0.2491i} & 0 \\ \end{array} } \right|$$

Here $\lambda = \pm \sqrt {2.9791 - 0.2491i}$. So, the point is unstable.
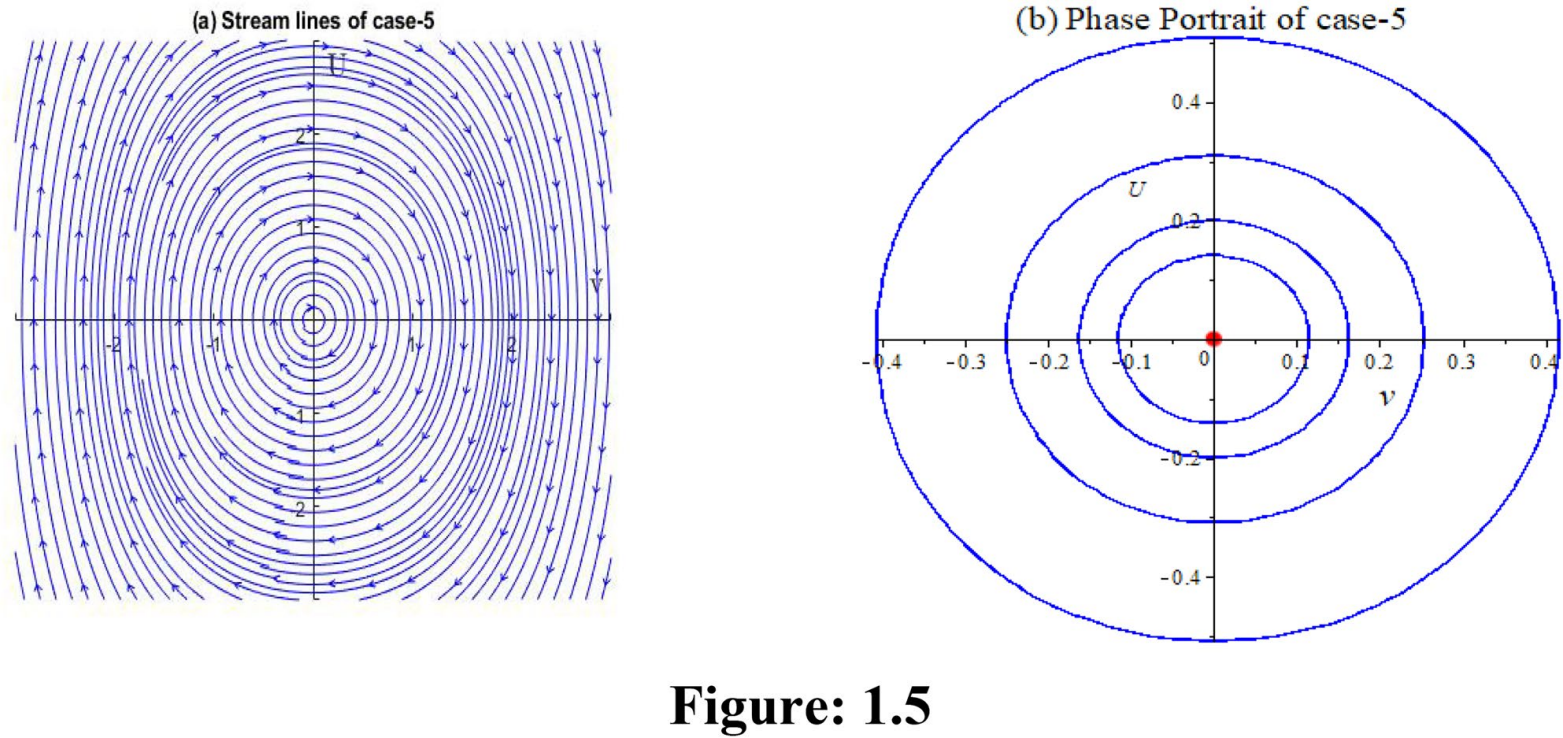


We get three numerical solutions for this case in Fig. 1.5 by setting three different initial points, resulting in three types of solutions. The initial points that we use are: (0.1667, 0), (0, 0.5), and (−0.2, −0.2).
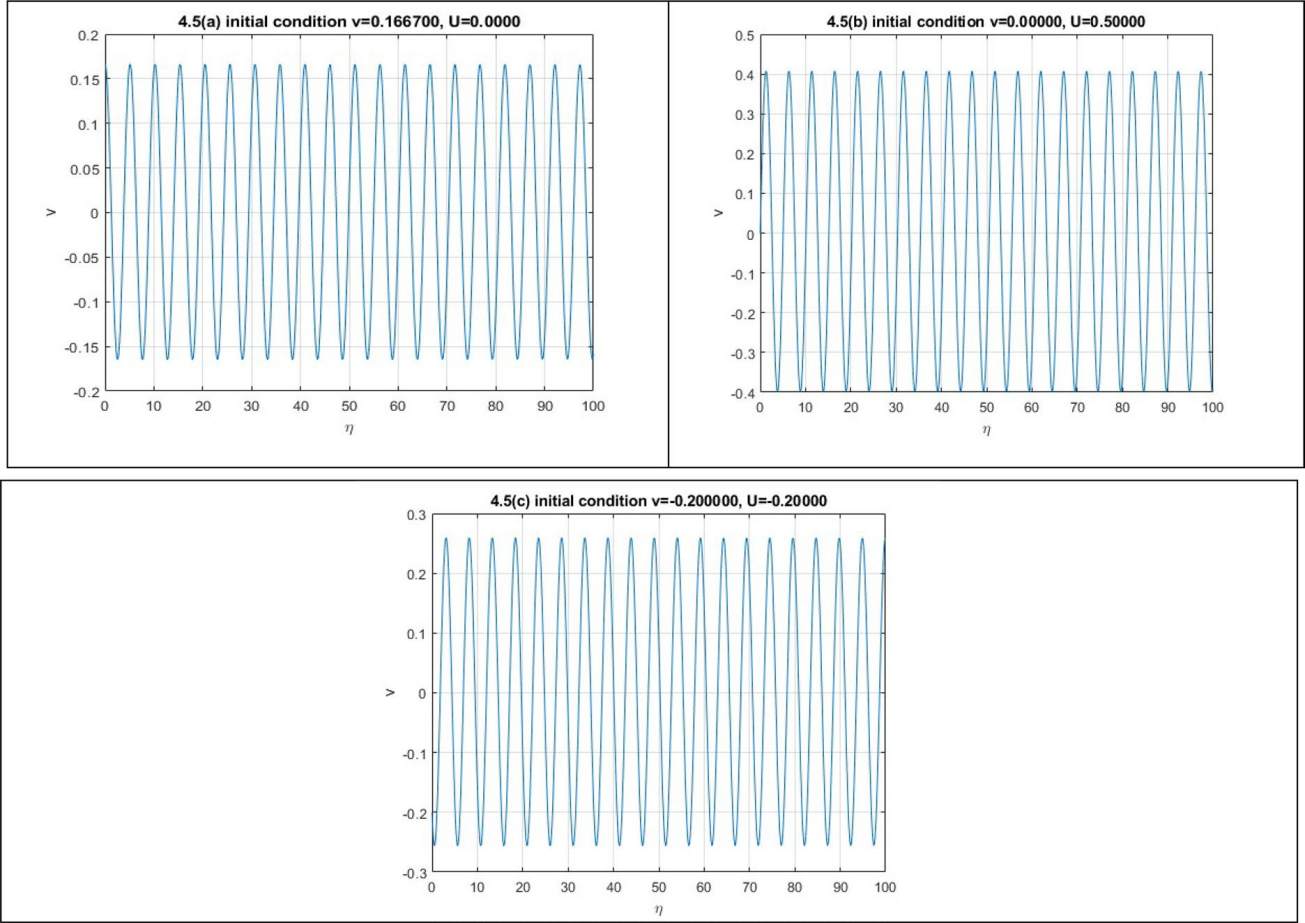


## Traveling wave solutions

From Eq. (5) we get,

6 $$\nu^{\prime\prime} = \frac{Q\nu }{P} - \frac{{R\nu^{2} }}{P} + \frac{{S\nu^{3} }}{P}$$

From (1.6), we establish a planar system as:

7 $$\left. \begin{gathered} \frac{dv}{{d\eta }} = U \hfill \\ \frac{dU}{{d\eta }} = \frac{Q}{P}\nu - \frac{R}{P}\nu^{2} + \frac{S}{P}\nu^{3} \hfill \\ \end{gathered} \right\}$$

The system (7) is a Hamiltonian system with the following Hamiltonian function:

$$H(\nu ,U) = \frac{{U^{2} }}{2} - \frac{Q}{2P}\nu^{2} + \frac{R}{3P}\nu^{3} - \frac{S}{4P}\nu^{4} = h$$

where $h$ is Hamiltonian.

$$\Rightarrow \frac{{U^{2} }}{2} = h + \frac{Q}{2P}\nu^{2} - \frac{R}{3P}\nu^{3} + \frac{S}{4P}\nu^{4}$$

$$\Rightarrow U = \pm \sqrt 2 \sqrt {h + \frac{Q}{2P}\nu^{2} - \frac{R}{3P}\nu^{3} + \frac{S}{4P}\nu^{4} }$$

8 $$\Rightarrow U = \pm \sqrt 2 \sqrt {K_{4} (\nu )}$$

$${\text{where}},\;\;K_{4} (\nu ) = h + \frac{Q}{2P}\nu^{2} - \frac{R}{3P}\nu^{3} + \frac{S}{4P}\nu^{4}$$

Now,

$$\frac{d\nu }{{d\eta }} = U$$

$$\Rightarrow \frac{d\nu }{U} = d\eta$$

9 $$\Rightarrow \frac{d\nu }{{ \pm \sqrt 2 \sqrt {K_{4} (\nu )} }} = d\eta$$

10 $$\Rightarrow \int {\frac{d\nu }{{ \pm \sqrt 2 \sqrt {K_{4} (\nu )} }} = \int {d\eta } }$$

Suppose that,

11 $$f(\nu ) = \frac{Q}{P}\nu - \frac{R}{P}\nu^{2} + \frac{S}{P}\nu^{3}$$

We know that the equation $f(\nu ) = 0$ has the following three real roots:

$$\nu_{0} = 0,\;\;\;\nu_{A} = \frac{R}{2S} + \frac{{\sqrt {R^{2} - 4QS} }}{2S},\;\;\nu_{B} = \frac{R}{2S} - \frac{{\sqrt {R^{2} - 4QS} }}{2S}$$

the function (11) has two extreme points

$$\nu_{ \pm }^{*} = \frac{{R \pm \sqrt {R^{2} - 3QS} }}{3S}$$

and two extreme values

$$f(\nu_{ \pm }^{*} ) = \frac{Q}{P}\left( {\frac{{R \pm \sqrt {R^{2} - 3QS} }}{3S}} \right) - \frac{R}{P}\left( {\frac{{R \pm \sqrt {R^{2} - 3QS} }}{3S}} \right)^{2} + \frac{S}{P}\left( {\frac{{R \pm \sqrt {R^{2} - 3QS} }}{3S}} \right)^{3}$$

## Case 1:

When, $$h = 0$$

$${\text{Then}},\;\;K_{4} (\nu ) = \nu^{2} \left( {\frac{Q}{2P} - \frac{R}{3P}\nu + \frac{S}{4P}\nu^{2} } \right)$$

Now from (9) we get,

$$\frac{d\nu }{{ \pm \sqrt 2 \nu \sqrt {\frac{Q}{2P} - \frac{R}{3P}\nu + \frac{S}{4P}\nu^{2} } }} = d\eta$$

$$\Rightarrow \frac{d\nu }{{\nu \left( {\nu - \frac{2R}{{3S}}} \right)}} = \pm \sqrt 2 d\eta$$

For discriminate, $$2R^{2} - 9SQ = 0,$$

$$\Rightarrow \frac{1}{{\frac{2R}{{3S}}}}\left( {\frac{1}{{\left( {\nu - \frac{2R}{{3S}}} \right)}} - \frac{1}{\nu }} \right)d\nu = \pm \sqrt 2 d\eta$$

$$\Rightarrow \int {\left( {\frac{1}{{\left( {\nu - \frac{2R}{{3S}}} \right)}} - \frac{1}{\nu }} \right)d\nu = \pm \sqrt 2 .\frac{2R}{{3S}}\int {d\eta } }$$

$$\Rightarrow \ln \left( {\nu - \frac{2R}{{3S}}} \right) - \ln \nu = \ln e^{{\left( { \pm \frac{2\sqrt 2 R}{{3S}}\eta } \right)}} + \ln c$$

$$\Rightarrow \ln \left( {\frac{{\nu - \frac{2R}{{3S}}}}{\nu }} \right) = \ln ce^{{\left( { \pm \frac{2\sqrt 2 R}{{3S}}\eta } \right)}}$$

$$\Rightarrow 1 - \frac{{\frac{2R}{{3S}}}}{\nu } = ce^{{\left( { \pm \frac{2\sqrt 2 R}{{3S}}\eta } \right)}}$$

$$\Rightarrow \nu = \frac{{\frac{2R}{{3S}}}}{{1 - ce^{{\left( {\frac{ \pm 2\sqrt 2 R}{{3S}}\eta } \right)}} }}$$

So, we get two kink wave solutions,

$$\nu_{ + } = \frac{2R}{{3S\left( {1 - ce^{{\frac{2\sqrt 2 R\eta }{{3S}}}} } \right)}}$$

$$\nu_{ - } = \frac{2R}{{3S\left( {1 - ce^{{\frac{ - 2\sqrt 2 R\eta }{{3S}}}} } \right)}}$$

In Fig. 1.1b, there are one homoclinic orbit $o_{1}$ and three special orbits $o_{2}$, $\underline{{o_{2} }}$ and $o_{3}$.The orbits are defined by $H(\nu ,U) = H(\nu_{B} ,0)$, which can be converted to

12 $$U = \pm \sqrt{\frac{S}{2P}} (\nu - \nu_{B} )\sqrt {(\nu - \nu_{1} )(\nu - \nu_{2} )}$$

where,

$$\nu_{B} = \frac{{R - \sqrt {R^{2} - 4QS} }}{2S}$$

$$\nu_{1} = \frac{{2R - 3rS + \sqrt {4R^{2} - 24RrS + 18r^{2} S^{2} + 18QS^{2} } }}{3S}$$

$$\nu_{2} = \frac{{2R - 3rS - \sqrt {4R^{2} - 24RrS + 18r^{2} S^{2} + 18QS^{2} } }}{3S}$$

Substituting (12) into $\frac{d\nu }{{d\eta }} = U$, we can obtain the following integrals by integrating along the aforementioned orbits.

$$\pm \int\limits_{{\nu_{1} }}^{\nu } {\frac{d\nu }{{(\nu - \nu_{B} )\sqrt {(\nu_{1} - \nu )(\nu_{2} - \nu )} }}} = \pm \sqrt{\frac{S}{2P}} \int\limits_{0}^{\eta } {d\eta } ,$$

$$\pm \int\limits_{\nu }^{ + \infty } {\frac{d\nu }{{(\nu - \nu_{B} )\sqrt {(\nu_{1} - \nu )(\nu_{2} - \nu )} }}} = \pm \sqrt{\frac{S}{2P}} \int\limits_{0}^{\eta } {d\eta } .$$

By completing the integrals, we obtain the solitary wave solution:

$$\nu = \frac{{\nu_{B} e^{{ \pm \sqrt {\frac{{2S(\nu_{B} - \nu_{1} )(\nu_{B} - \nu_{2} )}}{P}} t}} + 2(\nu_{B} \nu_{1} + \nu_{B} \nu_{2} - 2\nu_{1} \nu_{2} )e^{{^{{ \pm \sqrt {\frac{{S(\nu_{B} - \nu_{1} )(\nu_{B} - \nu_{2} )}}{2P}} t}} }} + \nu_{B} (\nu_{1} - \nu_{2} )}}{{e^{{ \pm \sqrt {\frac{{2S(\nu_{B} - \nu_{1} )(\nu_{B} - \nu_{2} )}}{P}} t}} + 2(2\nu_{B} - \nu_{1} - \nu_{2} )e^{{^{{ \pm \sqrt {\frac{{S(\nu_{B} - \nu_{1} )(\nu_{B} - \nu_{2} )}}{2P}} t}} }} + (\nu_{1} - \nu_{2} )}}$$

and the singular wave solution as:

$$\nu = \frac{{\nu_{B} e^{{ \pm \sqrt {\frac{{2S(\nu_{B} - \nu_{1} )(\nu_{B} - \nu_{2} )}}{P}} t}} + 2(\nu_{1} \nu_{2} - \nu_{B} \nu_{1} - \nu_{B} \nu_{2} )e^{{^{{ \pm \sqrt {\frac{{S(\nu_{B} - \nu_{1} )(\nu_{B} - \nu_{2} )}}{2P}} t}} }} + \nu_{B} (\nu_{1} - \nu_{2} )^{2} }}{{e^{{ \pm \sqrt {\frac{{2S(\nu_{B} - \nu_{1} )(\nu_{B} - \nu_{2} )}}{P}} t}} + 2(\nu_{1} + \nu_{2} - 2\nu_{B} )e^{{^{{ \pm \sqrt {\frac{{S(\nu_{B} - \nu_{1} )(\nu_{B} - \nu_{2} )}}{2P}} t}} }} + (\nu_{1} - \nu_{2} )^{2} }}$$

Similarly, there are three periodic orbits $o_{4} ,o_{5} ,\underline{{o_{5} }}$ and for which we get

13 $$U = \pm \sqrt{\frac{S}{2P}} \sqrt {(\nu - \nu_{7} )(\nu - \nu_{5} )(\nu - \nu_{6} )(\nu - \nu_{8} )}$$

Substituting (13) into $\frac{d\nu }{{d\eta }} = U$, and along the aforementioned orbits, we can obtain the following integrals as:

$$\pm \int\limits_{{\nu_{7} }}^{\nu } {\frac{dr}{{\sqrt {(r - \nu_{7} )(r - \nu_{5} )(r - \nu_{6} )(r - \nu_{8} )} }}} = \pm \sqrt{\frac{S}{2P}} \int\limits_{0}^{\eta } {d\eta } ,$$

$$\pm \int\limits_{{\nu_{6} }}^{\nu } {\frac{dr}{{\sqrt {(\nu_{7} - r)(\nu_{5} - r)(r - \nu_{6} )(r - \nu_{8} )} }}} = \pm \sqrt{\frac{S}{2P}} \int\limits_{0}^{\eta } {d\eta } ,$$

$$\pm \int\limits_{{\nu_{8} }}^{\nu } {\frac{dr}{{\sqrt {(\nu_{7} - r)(\nu_{5} - r)(\nu_{6} - r)(\nu_{8} - r)} }}} = \pm \sqrt{\frac{S}{2P}} \int\limits_{0}^{\eta } {d\eta } .$$

By completing the integrals, we get the periodic wave solutions as follows:

$$\nu = \frac{{\nu_{6} (\nu_{8} - \nu_{5} ) + \nu_{8} (\nu_{5} - \nu_{6} )sn^{2} \left( {\sqrt{\frac{S}{2P}} \frac{1}{{g_{2} }}\eta ,k_{2} } \right)}}{{\nu_{8} - \nu_{5} + (\nu_{5} - \nu_{6} )sn^{2} \left( {\sqrt{\frac{S}{2P}} \frac{1}{{g_{2} }}\eta ,k_{2} } \right)}},$$

$$\nu = \frac{{\nu_{8} (\nu_{6} - \nu_{7} ) + \nu_{6} (\nu_{7} - \nu_{8} )sn^{2} \left( {\sqrt{\frac{S}{2P}} \frac{1}{{g_{2} }}\eta ,k_{2} } \right)}}{{\nu_{6} - \nu_{7} + (\nu_{7} - \nu_{8} )sn^{2} \left( {\sqrt{\frac{S}{2P}} \frac{1}{{g_{2} }}\eta ,k_{2} } \right)}},$$

$$\nu = \frac{{\nu_{7} (\nu_{5} - \nu_{8} ) + \nu_{5} (\nu_{8} - \nu_{7} )sn^{2} \left( {\sqrt{\frac{S}{2P}} \frac{1}{{g_{2} }}\eta ,k_{2} } \right)}}{{\nu_{5} - \nu_{8} + (\nu_{8} - \nu_{7} )sn^{2} \left( {\sqrt{\frac{S}{2P}} \frac{1}{{g_{2} }}\eta ,k_{2} } \right)}}.$$

## Case 2:

In Fig. 1.2b, three special orbits $o_{8} ,\underline{{o_{8} }} ,o_{9}$ which are identify via $H(\nu ,U) = H(\nu_{10} ,0)$, yields

14 $$U = \pm \sqrt{\frac{S}{2P}} (\nu - \nu_{10} )\sqrt {(\nu - \nu_{10} )(\nu - \nu_{11} )}$$

where,

$$\nu_{10} = \frac{R}{2S},$$

$$\nu_{11} = - \frac{R}{6S}$$

Substituting (14) into $\frac{d\nu }{{d\eta }} = U$, and along the aforementioned orbits, we obtain the following integrals.

$$\pm \int\limits_{{\nu_{11} }}^{\nu } {\frac{dz}{{(z - \nu_{10} )\sqrt {(z - \nu_{10} )(z - \nu_{11} )} }}} = \pm \sqrt{\frac{S}{2P}} \int\limits_{0}^{\eta } {d\eta } ,$$

$$\pm \int\limits_{\nu }^{\infty } {\frac{dz}{{(z - \nu_{10} )\sqrt {(z - \nu_{10} )(z - \nu_{11} )} }}} = \pm \sqrt{\frac{S}{2P}} \int\limits_{0}^{\eta } {d\eta } .$$

By completing the integrals, we get the following wave solutions as:

$$\nu = \frac{{\nu_{11} + \nu_{10} }}{2} + \frac{{\nu_{10} - \nu_{11} }}{2}\left\{ {\frac{{\left( { \pm \sqrt{\frac{S}{2P}} \frac{{\nu_{11} - \nu_{10} }}{2}\eta } \right)^{2} + 1}}{{\left( { \pm \sqrt{\frac{S}{2P}} \frac{{\nu_{11} - \nu_{10} }}{2}\eta } \right)^{2} - 1}}} \right\},$$

$$\nu = \frac{{\nu_{11} + \nu_{10} }}{2} + \frac{{\nu_{10} - \nu_{11} }}{2}\left\{ {\frac{{\left( {1 \pm \sqrt{\frac{S}{2P}} \frac{{\nu_{11} - \nu_{10} }}{2}\eta } \right)^{2} + 1}}{{\left( {1 \pm \sqrt{\frac{S}{2P}} \frac{{\nu_{11} - \nu_{10} }}{2}\eta } \right)^{2} - 1}}} \right\}.$$

## Strengths, weaknesses, results, and discussion

### General strengths of the method

The method employed in our study, which combines Hamiltonian and Jacobian techniques within the framework of planar dynamical systems, offers several notable strengths.

First and foremost, it provides a comprehensive and systematic approach to analyzing nonlinear telecommunications models. By integrating Hamiltonian principles, we harness the power of energy conservation, allowing for a clear interpretation of system behavior in terms of energy orbits. This not only facilitates a deeper understanding of the underlying dynamics but also provides a valuable tool for engineers and researchers to monitor energy-related aspects in their own problems.

Secondly, the use of Jacobian techniques enables us to assess the stability of critical points within the model. This is of paramount importance in practical applications, as it allows for the prediction and management of system transitions and instabilities. Engineers can leverage this aspect of the method to optimize the stability of telecommunications networks, ensuring reliable performance.

Furthermore, our approach excels in deriving a wide range of wave solutions, from solitons to periodic waves, providing flexibility in representing different signal propagation scenarios. This versatility is a significant asset for those designing communication systems where signal types and characteristics vary.

### General weaknesses of the method

While our method offers many advantages, it also has certain limitations that readers should be aware of when considering its application.

One notable limitation is the potential complexity of the mathematical framework involved. The combination of Hamiltonian and Jacobian techniques may require a strong mathematical background, which could pose a challenge for those without extensive mathematical expertise. However, we have strived to make our approach as accessible as possible through clear explanations and illustrative examples.

Another consideration is the computational intensity associated with numerical simulations. Depending on the problem's complexity and the available computing resources, running simulations can be time-consuming and resource-intensive. Engineers and researchers should be prepared for this aspect and allocate sufficient computational resources accordingly.

In conclusion, our method offers a powerful and versatile approach for analyzing nonlinear telecommunications models, with strengths in energy conservation, critical point stability assessment, and wave solution derivation. While it may involve some complexity and computational demands, its practical utility is evident in its ability to provide insights into system behavior, stability, and signal propagation. We hope that these insights into the method's strengths and weaknesses will assist readers in applying it effectively to their own problems in the telecommunications domain.

## Results

In this section, we present the key findings and results of our study, which encompasses the analysis of bifurcation, stability, and wave solutions in a nonlinear model related to the telecommunications industry. Our investigation utilizes Hamiltonian and Jacobian techniques to provide a comprehensive understanding of the system's behavior.

1. Bifurcation Analysis: We commenced our study by analyzing the bifurcation behavior of the nonlinear telecommunications model. Through careful examination of the system's equations and parameters, we identified critical points and explored their stability. The results revealed various bifurcation scenarios, including saddle-node, pitchfork, and transcritical bifurcations, depending on specific parameter values.
2. Stability of Critical Points: To gain insights into the stability of the critical points, we employed graphical representations and numerical solutions. Our findings indicated that the system's stability is highly dependent on the parameter values. Certain parameter combinations led to stable critical points, while others resulted in unstable behavior, manifesting as limit cycles or chaotic dynamics.
3. Wave Solutions: One of the central aspects of our study was the derivation of wave solutions for the nonlinear telecommunications model. We explored both numerical and analytic solutions, guided by the energy orbits within the corresponding phase portraits. Our results showcased a wide range of wave behaviors, including solitons, periodic waves, and other nonlinear waveforms, depending on the initial conditions and parameter values.
4. Traveling Wave Solutions: In addition to static wave solutions, we investigated traveling wave solutions within the model. By analyzing the propagation of waves through the system, we identified conditions under which traveling wave behavior emerges. This analysis contributes to a deeper understanding of the dynamic aspects of the model.

## Discussion

In the discussion section, we analyze our results' implications and significance in telecommunications and nonlinear dynamics, offering insights and broader context:

1. Bifurcation Patterns and Telecommunications: Our identification of various bifurcation patterns in the nonlinear telecommunications model sheds light on the system's complex behavior. Understanding these patterns is crucial for telecommunications engineers and researchers as it helps predict and manage system transitions and instabilities.
2. Critical Point Stability and System Control: The stability analysis of critical points is essential for system control and optimization in telecommunications networks. Our findings highlight the sensitivity of system stability to parameter variations, emphasizing the need for careful parameter tuning to maintain a stable network.
3. Diverse Wave Solutions: The derivation of diverse wave solutions demonstrates the versatility of the nonlinear model in representing different types of signal propagation in telecommunications. These solutions have practical applications in designing communication systems capable of accommodating a variety of signal types and characteristics.
4. Traveling Wave Insights: Our investigation into traveling wave solutions enriches our understanding of wave propagation in the telecommunications context. It provides valuable insights for optimizing signal transmission over long distances and through complex networks.
5. Planar Dynamical Approach: Our results indicate that the planar dynamical approach is well-suited for capturing the behavior of the nonlinear telecommunications model. Its ability to provide comprehensive results following each orbit of the phase portraits is particularly advantageous for understanding the system's dynamics.

Our study offers a thorough analysis of a nonlinear model relevant to the telecommunications industry, with implications for network stability, signal propagation, and system design. The diverse range of results presented here contributes to advancing our understanding of complex systems in telecommunications and nonlinear dynamics.

## Comparative analysis

In this section, we assess the performance of our method alongside other recent existing methods, highlighting their respective strengths and weaknesses.

### Comparison with existing methods

Advantages of Our Method:

1. Comprehensive Analysis: Our method offers a comprehensive analysis of the nonlinear telecommunications model, covering bifurcation, stability, and wave solutions, which provides a holistic understanding of the system's behavior.
2. Use of Hamiltonian and Jacobian Techniques: The employment of Hamiltonian and Jacobian techniques allows for a more in-depth exploration of the model, enabling us to uncover various aspects of system dynamics.
3. Diverse Wave Solutions: Our study successfully derives a wide range of wave solutions, including solitons, periodic waves, and nonlinear waveforms, showcasing the versatility of the model in representing different signal propagation scenarios.
4. Traveling Wave Insights: In addition to static wave solutions, our analysis extends to traveling wave solutions, enhancing our understanding of wave propagation dynamics in telecommunications.
5. Planar Dynamical Approach: The planar dynamical approach, as utilized in our study, proves effective in capturing system behavior in alignment with phase portrait orbits, contributing to a deeper understanding of the model.

Disadvantages of Our Method:

1. Complexity: The comprehensive nature of our analysis may lead to a more complex mathematical framework, which could be challenging for some readers to grasp without a strong mathematical background.
2. Computational Intensity: The use of numerical methods and simulations in our analysis might require substantial computational resources, potentially limiting the applicability of our approach in resource-constrained environments.

Advantages of Existing Methods^44–52^:

1. Simplicity: Some existing methods in the field may offer simpler and more straightforward mathematical formulations, making them accessible to a broader audience.
2. Efficiency: Certain methods^44–52^ may be computationally more efficient, suitable for real-time or large-scale applications in the telecommunications industry.

Disadvantages of Existing Methods:

1. Scope: Some existing methods may focus on some quite aspects of the problem (e.g., bifurcation analysis or wave solutions), which may not provide a holistic view of the system's behavior.
2. Versatility: Other methods may offer quite versatility in representing various types of wave solutions, limiting their applicability to diverse telecommunications scenarios.

In conclusion, our method excels in providing a comprehensive analysis of the nonlinear telecommunications model, offering a wide range of wave solutions and insights into system dynamics. While it may have some complexity and computational intensity, these drawbacks are balanced by the depth of understanding it provides. Existing methods, on the other hand, may have their advantages in terms of simplicity and efficiency but may fall short in terms of scope and versatility. Researchers and practitioners can choose the most suitable method based on their specific needs and resources.

## Conclusion

In this research, we conducted an extensive examination of a nonlinear model relevant to the telecommunications sector. We employed Hamiltonian and Jacobian methods to derive numerical and analytical solutions, with a particular focus on aligning them with the energy orbits depicted in phase portraits. Our investigation also included the assessment of critical point stability through both graphical and numerical approaches, along with an exploration of traveling wave solutions. The graphical illustrations vividly demonstrated how parameters exert a significant influence on wave solutions, thereby highlighting the suitability of the planar dynamical approach for yielding comprehensive results in harmony with phase portrait orbits. In this study, we embarked on a comprehensive exploration of a nonlinear telecommunications model, employing Hamiltonian and Jacobian techniques to dissect its intricate behavior. Our journey through bifurcation analysis unearthed a tapestry of scenarios, from saddle-node to pitchfork and transcritical bifurcations, each offering unique insights into system transitions. The stability of critical points, a crucial consideration for network engineers, was scrutinized, emphasizing the paramount role of parameter tuning in achieving network stability.

The heart of our study lay in the derivation of wave solutions, both numerical and analytical, harmonizing with energy orbits depicted in phase portraits. This symphony of solutions unveiled a spectrum of wave behaviors, from solitons to periodic waves, demonstrating the model's adaptability in representing diverse signal propagation phenomena. Furthermore, our investigation delved into the realm of traveling wave solutions, shedding light on the dynamic aspects of wave propagation through telecommunications networks.

Throughout this voyage, the planar dynamical approach proved its worth, offering a holistic understanding of system behavior in alignment with phase portrait orbits. The elegance of this approach lies in its ability to traverse the intricate landscape of nonlinear dynamics, providing a clear path to comprehend complex system behaviors.

As we conclude our exploration, the implications of our findings resonate across the telecommunications landscape. Bifurcation patterns offer predictive insights for system transitions, critical point stability becomes the bedrock of network control, diverse wave solutions facilitate versatile signal design, and traveling wave insights empower long-distance signal transmission optimization.

Innovation thrives on understanding, and our study has illuminated the intricate dance of nonlinear dynamics within the telecommunications sector. We hope that our work not only serves as a valuable resource for engineers and researchers but also inspires further innovation in this ever-evolving field. As we peer into the horizon of telecommunications, armed with the knowledge derived from this study, we anticipate a future enriched with stable networks, diverse signals, and optimized transmission.

## Data Availability

All data generated or analyzed during this study are included in this article. No permissions are required from third-party.
